# ‘Foxtrot’ fumarate: a water-soluble salt of *N*,*N*-di­allyl-5-methoxytryptamine (5-MeO-DALT)

**DOI:** 10.1107/S2056989021002838

**Published:** 2021-03-19

**Authors:** Duyen N. K. Pham, Vamshikrishna Reddy Sammeta, Andrew R. Chadeayne, James A. Golen, David R. Manke

**Affiliations:** a University of Massachusetts Dartmouth, 285 Old Westport Road, North Dartmouth, MA 02747, USA; b CaaMTech, Inc., 58 East Sunset Way, Suite 209, Issaquah, WA 98027, USA

**Keywords:** crystal structure, tryptamines, indoles, hydrogen bonding

## Abstract

The title compound, bis­(*N*,*N*-diallyl-5-meth­oxy­tryptammonium) (5-MeO-DALT) fumarate (systematic name: bis­{*N*-[2-(5-meth­oxy-1*H*-indol-3-yl)eth­yl]- *N*-(prop-2-en-1-yl)prop-2-en-1-aminium} (*E*)-but-2-enedioate), 2C_17_H_23_N_2_O^+^·C_4_H_2_O_4_^2−^, has a single tryptammonium cation and half of a fumarate dianion in the asymmetric unit. The tryptammonium and fumarate ions are held together in one-dimensional chains by a series of N—H⋯O hydrogen bonds. These chains are combinations of *R* ^4^_4_(22) rings, and *C* ^2^_2_(14) and *C* ^4^_4_(28) parallel chains along [111].

## Abstract 2

The synthesis and solid-state structure of the fumarate salt of the synthetic psychedelic 5-meth­oxy-*N*,*N*-di­allyl­tryptamine (5-MeO-DALT) is reported.

## Chemical context   

Psychotropic compounds have gained a lot of attention in recent years for their potential as therapeutics to treat depression, anxiety, post-traumatic stress disorder, and addiction, among other disorders (Nichols & Hendricks, 2020 ▸). 5-Meth­oxy-*N*,*N*-di­methyl­tryptamine (5-MeO-DMT) is a naturally occurring tryptamine found in the parotid gland of some toads, and this compound has been explored for its clinical effects in treating mood disorders (Davis *et al.*, 2018 ▸). 5-MeO-DMT is highly active at the serotonin (5-hy­droxy­tryptamine, 5-HT) 2A receptor, which is the origin of its psychotropic activity. It can be administered *via* inhalation or injection, but does not function as a psychedelic when consumed orally (Weil & Davis, 1994 ▸). A recent report described the synthesis of a water-soluble succinate salt of 5-MeO-DMT (Sherwood *et al.*, 2020 ▸).
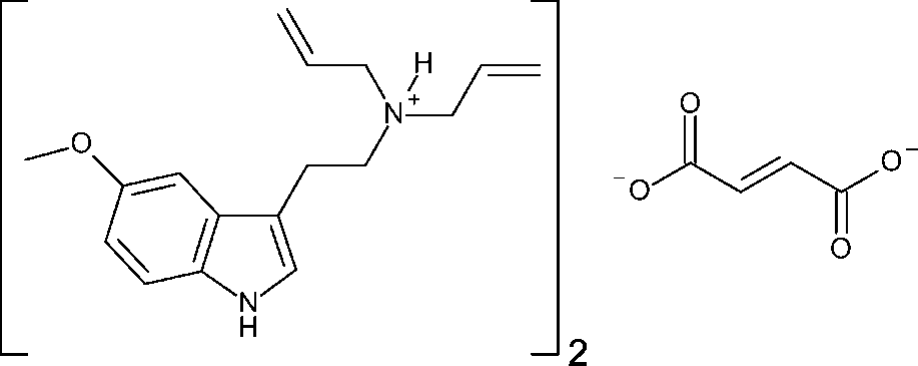


5-Meth­oxy-*N*,*N*-di­allyl­tryptamine (5-MeO-DALT) is a synthetic analogue of 5-MeO-DMT, which was synthesized in 2004 by Alexander Shulgin (Shulgin & Shulgin, 2016 ▸). The compound has potential as a therapeutic because it has a quick onset and rapid drop-off relative to other psychotropic tryptamines (Corkery *et al.*, 2012 ▸). Unlike 5-MeO-DMT, 5-MeO-DALT demonstrates activity when consumed orally, further improving its potential as a drug candidate. 5-MeO-DALT shows activity at a number of serotonin receptors, including 5-HT_1A_, 5-HT_1D_, 5-HT_2A_, 5-HT_2B_, 5-HT_6_ and 5-HT_7_ (Cozzi & Daley, 2016 ▸). As this class of mol­ecules become more significant in the treatment of mood disorders, it is important to have analytically pure, well-characterized, crystalline material to study the unique impact of individual compounds from the diverse range of compounds. It is also important to explore the effects of analytically pure combinations of these compounds to explore potential entourage effects. To best administer these compounds orally active, water-soluble crystalline materials are ideal. To that end, we set out to synthesize a water-soluble salt of 5-MeO-DALT, and report the synthesis and structure of bis­(5-meth­oxy-*N*,*N*-di­allyl­tryptammonium) fumarate herein.

## Structural commentary   

**Figure 1:**
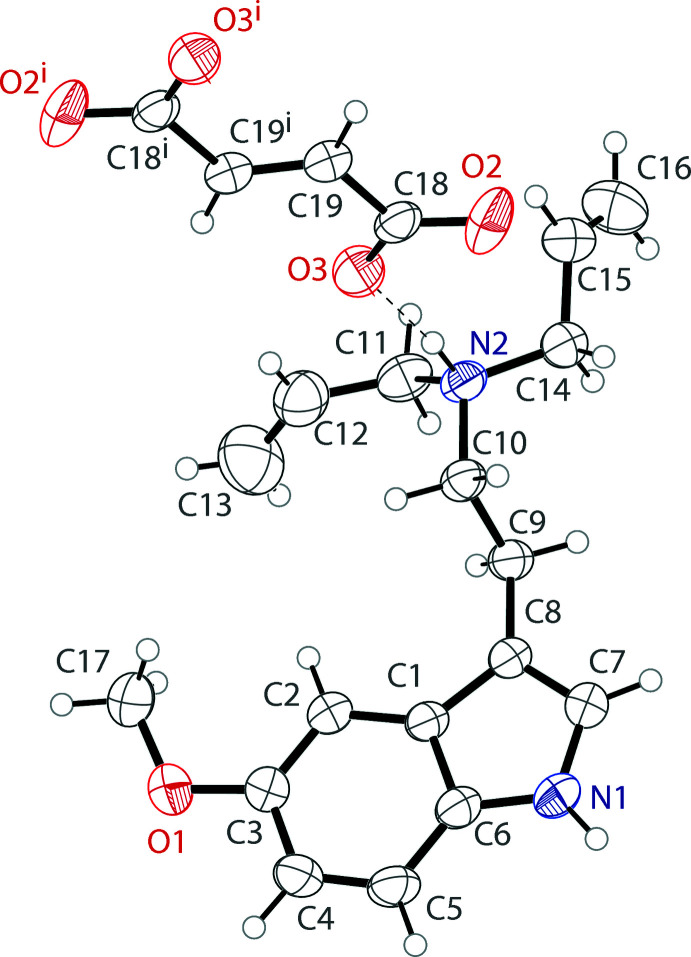
The mol­ecular structure of bis­(5-meth­oxy-*N*,*N*-di­allyl­tryptammonium) fumarate, showing the atom labelling. Displacement ellipsoids are drawn at the 50% probability level. Hydrogen bonds are shown as dashed lines. Symmetry code: (i) −*x*, 1 − *y*, −*z*.

The asymmetric unit of bis­(5-meth­oxy-*N*,*N*-di­allyl­tryptammonium) fumarate contains one tryptammonium cation and one half of a fumarate dianion (Fig. 1 ▸). The cation possesses a near planar indole ring, with a mean deviation from planarity of 0.011 Å. The meth­oxy group is turned slightly away from this plane, with a C2—C3—O1—C17 torsion angle of −13.9 (2)°. The ethyl­amino group is turned away from this plane, with a C7—C8—C9—C10 torsion angle of −103.9 (2)°. The second half of the fumarate dianion is generated by inversion, and the dianion is near planar, with a mean deviation from planarity of 0.057 Å. The carboxyl­ate unit is delocalized, with C—O distances of 1.271 (2) and 1.240 (2) Å. The nature of this salt allows for it to have high solubility in water, while the freebase does not.

## Supra­molecular features   

**Figure 2:**
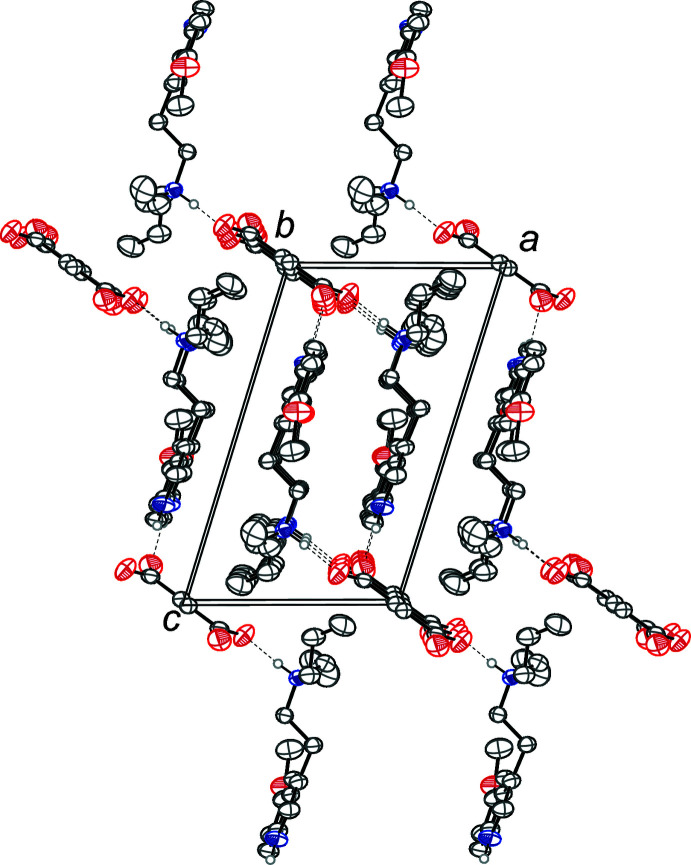
The crystal packing of bis­(5-meth­oxy-*N*,*N*-di­allyl­tryptammonium) fumarate, viewed along the *b* axis. The N—H⋯O hydrogen bonds (Table 1 ▸) are shown as dashed lines. Hydrogen atoms not involved in hydrogen bonding are omitted for clarity.

**Figure 3:**
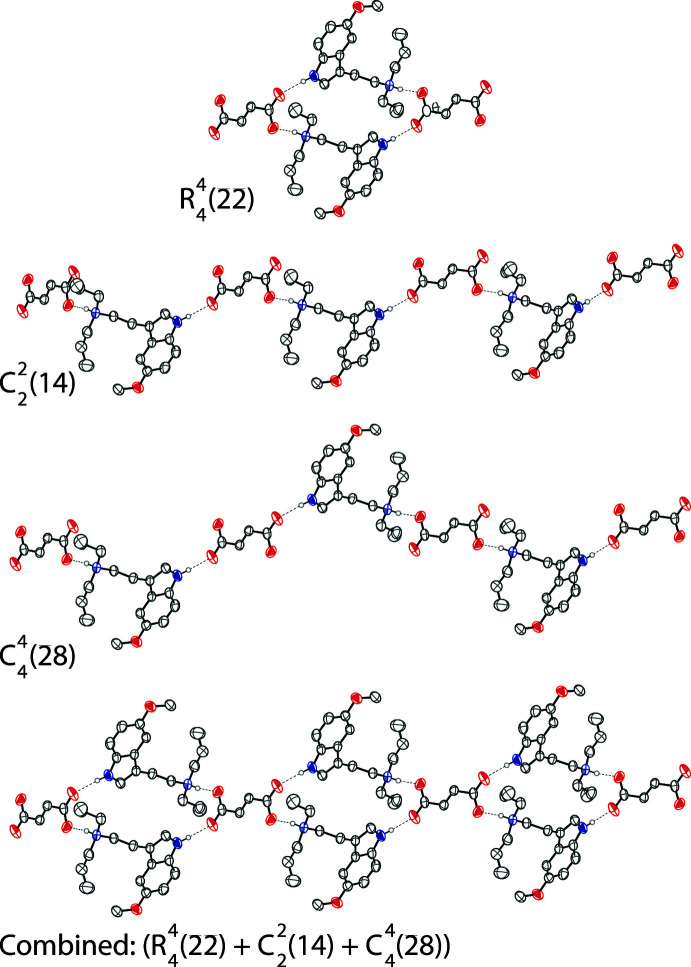
The hydrogen-bonding network along [111], which consists of 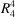
(22) rings that are joined together by two parallel 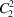
(14) and 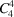
(28) chains. The three components described in graph-set notation and the combined chain are shown. Displacement ellipsoids are drawn at the 50% probability level. Hydrogen atoms not involved in hydrogen bonding are omitted for clarity. Hydrogen bonds are shown as dashed lines.

The tryptammonium cation and the fumarate dianion are linked together in the asymmetric unit through an N—H⋯O hydrogen bond between the ammonium nitro­gen and a carboxyl­ate oxygen (Table 1 ▸, Fig. 2 ▸). The indole nitro­gen also exhibits an N—H⋯O hydrogen bond with another symmetry generated fumarate dianion. Two tryptammonium cations and two fumarate dianions are joined together through the N—H⋯O hydrogen bonds to form rings with graph-set notation 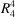
(22) (Etter *et al.*, 1990 ▸). The rings are joined together by two parallel chains along [111]. These chains have graph-set notation 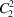
(14) and 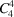
(28). The chains and rings are shown in Fig. 3 ▸. The hydrogen bond donor–acceptor distances of 2.5669 (16) Å and 2.7729 (17) Å indicate strong hydrogen bonds, with the N2—H2⋯O3 bond being stronger due to a charged donor and acceptor (Desiraju & Steiner, 2001 ▸).

## Database survey   

The structure of the freebase of 5-MeO-DALT has previously been reported (CCDC 1995802; Chadeayne *et al.*, 2020*d* ▸). The other tryptamine fumarate salts reported are those of 4-hy­droxy-*N*-methyl-*N*-iso­propyl­tryptamine (4-HO-MiPT) (TUFQAP; Chadeayne *et al.*, 2020*a* ▸), norpsilocin (4-HO-NMT) (MULXEZ; Chadeayne *et al.*, 2020*b* ▸), 4-acet­oxy-*N*,*N*-di­methyl­tryptamine (4-AcO-DMT) (XOFDOO; Chadeayne, Golen & Manke, 2019*a* ▸) and 4-hy­droxy-*N*,*N*-di-*n*-propyl­tryptamine (4-HO-DPT) (WUCGAF; Chadeayne, Pham *et al.*, 2019*b* ▸). There have also been a number of hydro­fumarate tryptamine salts reported, namely those of 4-AcO-DMT (HOCJUH; Chadeayne, Golen & Manke, 2019*b* ▸), *N*-methyl-*N*-iso­propyl­tryptamine (MiPT) and 4-HO-MiPT (RONSOF and RONSUL; Chadeayne, Pham *et al.*, 2019*a* ▸), *N*-ethyl-*N*-*n*-propyl­tryptamine (EPT) and *N*-methyl-*N*-allyl­tryptamine (MALT) (GUPBOL and GUPBUR; Chadeayne *et al.*, 2020*c* ▸). The MALT structure is the only other structure of an *N*-allyl tryptamine reported. There are a number of other 5-*O*-substituted tryptamines whose structures have been reported, including bufotenine (BUFTEN; Falkenberg, 1972 ▸), 5-MeO-DMT hydro­chloride (MOTYPT; Falkenberg & Carlström, 1971 ▸), 5-meth­oxy­tryptamine (MXTRUP; Quarles *et al.*, 1974 ▸), 5-MeO-DMT and 5-meth­oxy­mono­methyl­tryptamine (QQQAGY and QQQAHA; Bergin *et al.*, 1968 ▸). Three 2-Me-substituted 5-MeO-tryptamines were recently reported (CCDC 2058143, 2058144, 2058145; Pham *et al.* 2021 ▸).

## Synthesis and crystallization   

110 mg of 5-MeO-DALT freebase were dissolved in 10 mL of methanol and 47 mg of fumaric acid was added and refluxed overnight. 129 mg (82% yield) of white powder was obtained upon removal of solvent *in vacuo*. Single crystals suitable for X-ray diffraction were obtained by slow evaporation of an aqueous solution. The product was analysed by ^1^H NMR and ^13^C NMR. ^1^H NMR (400 MHz, D_2_O): δ 7.44 (*d*, *J* = 8.8 Hz, 1 H, Ar*H*), 7.27 (*s*, 1 H, Ar*H*), 7.10 (*d*, *J* = 2.3 Hz, 1 H, Ar*H*), 6.94 (*dd*, *J* = 8.8, 2.4 Hz, 1 H, Ar*H*), 6.67 (*s*, 2 H, C*H*), 5.91–5.81 (*m*, 2 H, C*H*), 5.62–5.56 (*m*, 4 H, C*H*_2_), 3.87 (*s*, 3 H, C*H*_3_), 3.79 (*d*, *J* = 7.2 Hz, 4 H, C*H*_2_), 3.42–3.38 (*m*, 2 H, C*H*_2_), 3.17–3.13 (*m*, 2 H, C*H*_2_); ^13^C NMR (100 MHz, D_2_O): δ 172.1 (*C*OO), 152.7 (*C*H), 135.3 (Ar*C*), 132.5 (Ar*C*), 127.22 (Ar*C*), 127.20 (Ar*C*), 126.2 (Ar*C*), 125.8 (Ar*C*), 113.7 (Ar*C*), 112.6 (Ar*C*), 108.9 (*C*H=CH_2_), 101.3 (CH=*C*H_2_), 56.8 (Ak*C*), 55.7 (Ak*C*), 52.2 (Ak*C*), 20.4 (Ak*C*).

## Refinement   

Crystal data, data collection and structure refinement details are summarized in Table 2 ▸. The hydrogen atoms on the indole nitro­gen (H1), and the amine (H2), were found in a difference-Fourier map and were refined isotropically, using DFIX restraints with N—H distances of 0.87 (1) Å. Isotropic displacement parameters were set to 1.2*U*_eq_ of the parent nitro­gen atom. All other hydrogen atoms were placed in calculated positions (C—H = 0.93–0.97 Å). Isotropic displacement parameters were set to 1.2*U*_eq_ (CH,CH_2_) or 1.5*U*_eq_ (CH_3_).

## Supplementary Material

Crystal structure: contains datablock(s) I. DOI: 10.1107/S2056989021002838/ey2005sup1.cif

Structure factors: contains datablock(s) I. DOI: 10.1107/S2056989021002838/ey2005Isup2.hkl

Supporting information file. DOI: 10.1107/S2056989021002838/ey2005Isup3.cml

CCDC reference: 2070873

Additional supporting information:  crystallographic information; 3D view; checkCIF report

## Figures and Tables

**Table 1:** Hydrogen-bond geometry (Å, °)

*D*—H⋯*A*	*D*—H	H⋯*A*	*D*⋯*A*	*D*—H⋯*A*
N1—H1⋯O2^i^	0.87 (1)	1.91 (1)	2.7729 (17)	175 (2)
N2—H2⋯O3	0.90 (1)	1.68 (1)	2.5669 (16)	171 (2)

Symmetry code: (i) -x+1, -y+2, -z+1.

**Table 2:** Experimental details

Crystal data	
Chemical formula	C_17_H_23_N_2_O^+^·0.5C_4_H_2_O_4_^2−^
*M*_r_	328.40
Crystal system, space group	Triclinic, *P*\overline{1}
Temperature (K)	297
*a*, *b*, *c* (Å)	7.8791 (7), 9.2908 (7), 13.5352 (11)
α, β, γ (°)	108.081 (3), 104.365 (3), 95.903 (3)
*V* (Å^3^)	894.87 (13)
*Z*	2
Radiation type	Mo *K*α
μ (mm^−1^)	0.08
Crystal size (mm)	0.34 × 0.28 × 0.22

Data collection	
Diffractometer	Bruker D8 Venture CMOS
Absorption correction	Multi-scan (*SADABS*; Bruker, 2018 ▸)
*T*_min_, *T*_max_	0.711, 0.745
No. of measured, independent and observed [*I* > 2σ(*I*)] reflections	27913, 3383, 2788
*R*_int_	0.035
(sin θ/λ)_max_ (Å^−1^)	0.611

Refinement	
*R*[*F* ^2^ > 2σ(*F* ^2^)], *wR*(*F* ^2^), *S*	0.043, 0.114, 1.05
No. of reflections	3383
No. of parameters	226
No. of restraints	2
H-atom treatment	H atoms treated by a mixture of independent and constrained refinement
Δρ_max_, Δρ_min_ (e Å^−3^)	0.26, −0.16

Computer programs: *APEX3* and *SAINT* (Bruker, 2018 ▸), SHELXT2014 (Sheldrick, 2015*a* ▸), *SHELXL2018* (Sheldrick, 2015*b* ▸), *OLEX2* (Dolomanov *et al.*, 2009 ▸) and *publCIF* (Westrip, 2010 ▸).

## supplementary crystallographic information

### Crystal data

**Table:**

C_17_H_23_N_2_O^+^·0.5C_4_H_2_O_4_^2^^−^	*Z* = 2
*M**_r_* = 328.40	*F*(000) = 352
Triclinic, *P*1	*D*_x_ = 1.219 Mg m^−^^3^
*a* = 7.8791 (7) Å	Mo *K*α radiation, λ = 0.71073 Å
*b* = 9.2908 (7) Å	Cell parameters from 9847 reflections
*c* = 13.5352 (11) Å	θ = 2.7–25.7°
α = 108.081 (3)°	µ = 0.08 mm^−^^1^
β = 104.365 (3)°	*T* = 297 K
γ = 95.903 (3)°	Block, orange
*V* = 894.87 (13) Å^3^	0.34 × 0.28 × 0.22 mm

### Data collection

**Table:**

Bruker D8 Venture CMOS diffractometer	2788 reflections with *I* > 2σ(*I*)
φ and ω scans	*R*_int_ = 0.035
Absorption correction: multi-scan (SADABS; Bruker, 2018)	θ_max_ = 25.7°, θ_min_ = 2.7°
*T*_min_ = 0.711, *T*_max_ = 0.745	*h* = −9→9
27913 measured reflections	*k* = −11→11
3383 independent reflections	*l* = −16→16

### Refinement

**Table:**

Refinement on *F*^2^	Primary atom site location: dual
Least-squares matrix: full	Hydrogen site location: mixed
*R*[*F*^2^ > 2σ(*F*^2^)] = 0.043	H atoms treated by a mixture of independent and constrained refinement
*wR*(*F*^2^) = 0.114	*w* = 1/[σ^2^(*F*_o_^2^) + (0.0485*P*)^2^ + 0.266*P*] where *P* = (*F*_o_^2^ + 2*F*_c_^2^)/3
*S* = 1.05	(Δ/σ)_max_ = 0.001
3383 reflections	Δρ_max_ = 0.26 e Å^−^^3^
226 parameters	Δρ_min_ = −0.16 e Å^−^^3^
2 restraints	

### Special details

**Table:**

Geometry. All esds (except the esd in the dihedral angle between two l.s. planes) are estimated using the full covariance matrix. The cell esds are taken into account individually in the estimation of esds in distances, angles and torsion angles; correlations between esds in cell parameters are only used when they are defined by crystal symmetry. An approximate (isotropic) treatment of cell esds is used for estimating esds involving l.s. planes.

### Fractional atomic coordinates and isotropic or equivalent isotropic displacement parameters (Å^2^)

**Table:**

	*x*	*y*	*z*	*U*_iso_*/*U*_eq_	
O3	0.32558 (15)	0.59832 (14)	0.09859 (10)	0.0582 (3)	
O2	0.21841 (18)	0.81313 (14)	0.10656 (11)	0.0675 (4)	
O1	0.7398 (2)	0.32650 (13)	0.56660 (10)	0.0647 (4)	
N1	0.7725 (2)	0.95161 (15)	0.70532 (10)	0.0491 (3)	
H1	0.773 (2)	1.0212 (17)	0.7654 (10)	0.058 (5)*	
N2	0.63718 (16)	0.74775 (14)	0.22313 (9)	0.0392 (3)	
H2	0.5248 (15)	0.704 (2)	0.1812 (15)	0.078 (6)*	
C1	0.77565 (19)	0.72996 (16)	0.57820 (11)	0.0372 (3)	
C2	0.7682 (2)	0.57053 (17)	0.53501 (11)	0.0412 (3)	
H2A	0.774806	0.524400	0.464848	0.049*	
C3	0.7507 (2)	0.48447 (17)	0.59951 (12)	0.0453 (4)	
C4	0.7447 (2)	0.55271 (19)	0.70629 (13)	0.0503 (4)	
H4	0.735505	0.491381	0.748234	0.060*	
C5	0.7522 (2)	0.70801 (19)	0.74980 (12)	0.0494 (4)	
H5	0.747839	0.753096	0.820581	0.059*	
C6	0.7667 (2)	0.79678 (17)	0.68493 (11)	0.0420 (3)	
C7	0.7879 (2)	0.98335 (18)	0.61541 (12)	0.0460 (4)	
H7	0.795238	1.080810	0.609612	0.055*	
C8	0.79099 (19)	0.85197 (16)	0.53544 (11)	0.0386 (3)	
C9	0.80805 (19)	0.83539 (17)	0.42450 (11)	0.0400 (3)	
H9A	0.894016	0.770455	0.409345	0.048*	
H9B	0.852886	0.936130	0.423723	0.048*	
C10	0.63041 (19)	0.76520 (17)	0.33583 (11)	0.0386 (3)	
H10A	0.587159	0.664307	0.336960	0.046*	
H10B	0.544568	0.829401	0.352776	0.046*	
C11	0.7478 (2)	0.63509 (19)	0.18188 (13)	0.0508 (4)	
H11A	0.750182	0.632850	0.110119	0.061*	
H11B	0.869410	0.668790	0.229759	0.061*	
C12	0.6761 (3)	0.4763 (2)	0.17570 (15)	0.0624 (5)	
H12	0.555703	0.436157	0.138805	0.075*	
C13	0.7660 (4)	0.3904 (3)	0.2168 (2)	0.0886 (7)	
H13A	0.886868	0.425891	0.254358	0.106*	
H13B	0.710420	0.292544	0.209010	0.106*	
C14	0.6880 (2)	0.90039 (18)	0.21243 (12)	0.0501 (4)	
H14A	0.623177	0.972370	0.248743	0.060*	
H14B	0.814472	0.939713	0.249493	0.060*	
C17	0.7066 (3)	0.2475 (2)	0.45399 (15)	0.0660 (5)	
H17A	0.673648	0.138566	0.438067	0.099*	
H17B	0.812447	0.268352	0.433750	0.099*	
H17C	0.611040	0.282080	0.413694	0.099*	
C15	0.6516 (3)	0.8949 (2)	0.09796 (13)	0.0570 (4)	
H15	0.538880	0.845917	0.049925	0.068*	
C16	0.7676 (3)	0.9543 (3)	0.06119 (18)	0.0783 (6)	
H16A	0.881409	1.004033	0.107318	0.094*	
H16B	0.737080	0.947286	−0.011317	0.094*	
C19	0.02229 (19)	0.57392 (16)	0.00887 (11)	0.0392 (3)	
H19	−0.063089	0.621325	−0.022769	0.047*	
C18	0.2012 (2)	0.67065 (17)	0.07656 (11)	0.0416 (3)	

### Atomic displacement parameters (Å^2^)

**Table:**

	*U*^11^	*U*^22^	*U*^33^	*U*^12^	*U*^13^	*U*^23^
O3	0.0431 (6)	0.0573 (7)	0.0615 (7)	−0.0031 (5)	−0.0059 (5)	0.0244 (6)
O2	0.0745 (9)	0.0416 (7)	0.0662 (8)	−0.0010 (6)	0.0246 (7)	−0.0080 (6)
O1	0.1008 (10)	0.0379 (6)	0.0513 (7)	0.0104 (6)	0.0145 (7)	0.0174 (5)
N1	0.0683 (9)	0.0402 (7)	0.0320 (6)	0.0074 (6)	0.0156 (6)	0.0039 (5)
N2	0.0384 (7)	0.0423 (7)	0.0301 (6)	0.0001 (5)	0.0070 (5)	0.0083 (5)
C1	0.0399 (7)	0.0378 (7)	0.0291 (6)	0.0026 (6)	0.0067 (6)	0.0096 (6)
C2	0.0487 (8)	0.0389 (8)	0.0314 (7)	0.0058 (6)	0.0097 (6)	0.0086 (6)
C3	0.0518 (9)	0.0386 (8)	0.0404 (8)	0.0040 (7)	0.0070 (7)	0.0137 (6)
C4	0.0587 (10)	0.0528 (10)	0.0397 (8)	0.0012 (7)	0.0100 (7)	0.0231 (7)
C5	0.0597 (10)	0.0556 (10)	0.0299 (7)	0.0036 (8)	0.0138 (7)	0.0127 (7)
C6	0.0478 (8)	0.0411 (8)	0.0308 (7)	0.0040 (6)	0.0094 (6)	0.0073 (6)
C7	0.0574 (9)	0.0367 (8)	0.0388 (8)	0.0033 (7)	0.0097 (7)	0.0115 (6)
C8	0.0423 (8)	0.0369 (7)	0.0311 (7)	0.0015 (6)	0.0064 (6)	0.0097 (6)
C9	0.0420 (8)	0.0421 (8)	0.0323 (7)	0.0006 (6)	0.0077 (6)	0.0132 (6)
C10	0.0393 (7)	0.0416 (8)	0.0316 (7)	0.0020 (6)	0.0111 (6)	0.0096 (6)
C11	0.0533 (9)	0.0577 (10)	0.0385 (8)	0.0129 (8)	0.0176 (7)	0.0089 (7)
C12	0.0685 (12)	0.0557 (11)	0.0576 (11)	0.0183 (9)	0.0149 (9)	0.0133 (9)
C13	0.0978 (18)	0.0812 (16)	0.0977 (17)	0.0381 (14)	0.0339 (14)	0.0358 (14)
C14	0.0596 (10)	0.0456 (9)	0.0391 (8)	−0.0012 (7)	0.0108 (7)	0.0134 (7)
C17	0.0925 (15)	0.0400 (9)	0.0539 (10)	0.0135 (9)	0.0097 (10)	0.0099 (8)
C15	0.0681 (11)	0.0579 (10)	0.0403 (8)	0.0039 (8)	0.0085 (8)	0.0191 (8)
C16	0.1000 (17)	0.0871 (15)	0.0617 (12)	0.0149 (13)	0.0341 (12)	0.0381 (11)
C19	0.0391 (7)	0.0439 (7)	0.0314 (7)	0.0085 (6)	0.0087 (6)	0.0097 (6)
C18	0.0472 (8)	0.0436 (8)	0.0272 (7)	0.0006 (7)	0.0120 (6)	0.0049 (6)

### Geometric parameters (Å, º)

**Table:**

O3—C18	1.2709 (19)	C9—H9B	0.9700
O2—C18	1.2400 (19)	C9—C10	1.5224 (19)
O1—C3	1.3817 (19)	C10—H10A	0.9700
O1—C17	1.414 (2)	C10—H10B	0.9700
N1—H1	0.865 (9)	C11—H11A	0.9700
N1—C6	1.372 (2)	C11—H11B	0.9700
N1—C7	1.368 (2)	C11—C12	1.494 (3)
N2—H2	0.897 (9)	C12—H12	0.9300
N2—C10	1.4982 (17)	C12—C13	1.282 (3)
N2—C11	1.494 (2)	C13—H13A	0.9300
N2—C14	1.494 (2)	C13—H13B	0.9300
C1—C2	1.402 (2)	C14—H14A	0.9700
C1—C6	1.4073 (19)	C14—H14B	0.9700
C1—C8	1.431 (2)	C14—C15	1.488 (2)
C2—H2A	0.9300	C17—H17A	0.9600
C2—C3	1.375 (2)	C17—H17B	0.9600
C3—C4	1.402 (2)	C17—H17C	0.9600
C4—H4	0.9300	C15—H15	0.9300
C4—C5	1.367 (2)	C15—C16	1.297 (3)
C5—H5	0.9300	C16—H16A	0.9300
C5—C6	1.394 (2)	C16—H16B	0.9300
C7—H7	0.9300	C19—C19^i^	1.312 (3)
C7—C8	1.364 (2)	C19—H19	0.9300
C8—C9	1.5025 (19)	C19—C18	1.491 (2)
C9—H9A	0.9700		
			
C3—O1—C17	116.62 (13)	N2—C10—H10A	108.6
C6—N1—H1	127.5 (13)	N2—C10—H10B	108.6
C7—N1—H1	123.8 (13)	C9—C10—H10A	108.6
C7—N1—C6	108.60 (12)	C9—C10—H10B	108.6
C10—N2—H2	103.9 (13)	H10A—C10—H10B	107.5
C11—N2—H2	105.0 (13)	N2—C11—H11A	109.3
C11—N2—C10	113.75 (12)	N2—C11—H11B	109.3
C14—N2—H2	109.2 (13)	H11A—C11—H11B	107.9
C14—N2—C10	111.74 (11)	C12—C11—N2	111.77 (14)
C14—N2—C11	112.46 (13)	C12—C11—H11A	109.3
C2—C1—C6	119.75 (13)	C12—C11—H11B	109.3
C2—C1—C8	133.20 (13)	C11—C12—H12	117.1
C6—C1—C8	107.05 (12)	C13—C12—C11	125.7 (2)
C1—C2—H2A	121.0	C13—C12—H12	117.1
C3—C2—C1	118.00 (13)	C12—C13—H13A	120.0
C3—C2—H2A	121.0	C12—C13—H13B	120.0
O1—C3—C4	114.40 (14)	H13A—C13—H13B	120.0
C2—C3—O1	123.88 (14)	N2—C14—H14A	108.8
C2—C3—C4	121.71 (14)	N2—C14—H14B	108.8
C3—C4—H4	119.5	H14A—C14—H14B	107.6
C5—C4—C3	121.06 (14)	C15—C14—N2	114.01 (13)
C5—C4—H4	119.5	C15—C14—H14A	108.8
C4—C5—H5	121.0	C15—C14—H14B	108.8
C4—C5—C6	118.05 (14)	O1—C17—H17A	109.5
C6—C5—H5	121.0	O1—C17—H17B	109.5
N1—C6—C1	107.68 (13)	O1—C17—H17C	109.5
N1—C6—C5	130.91 (14)	H17A—C17—H17B	109.5
C5—C6—C1	121.41 (14)	H17A—C17—H17C	109.5
N1—C7—H7	124.8	H17B—C17—H17C	109.5
C8—C7—N1	110.47 (14)	C14—C15—H15	118.1
C8—C7—H7	124.8	C16—C15—C14	123.87 (18)
C1—C8—C9	125.93 (13)	C16—C15—H15	118.1
C7—C8—C1	106.20 (13)	C15—C16—H16A	120.0
C7—C8—C9	127.88 (14)	C15—C16—H16B	120.0
C8—C9—H9A	109.2	H16A—C16—H16B	120.0
C8—C9—H9B	109.2	C19^i^—C19—H19	118.0
C8—C9—C10	112.05 (12)	C19^i^—C19—C18	124.09 (18)
H9A—C9—H9B	107.9	C18—C19—H19	118.0
C10—C9—H9A	109.2	O3—C18—C19	116.27 (13)
C10—C9—H9B	109.2	O2—C18—O3	125.12 (14)
N2—C10—C9	114.87 (11)	O2—C18—C19	118.62 (15)
			
O1—C3—C4—C5	−179.30 (16)	C6—C1—C8—C9	178.77 (14)
N1—C7—C8—C1	0.28 (18)	C7—N1—C6—C1	−1.02 (18)
N1—C7—C8—C9	−179.37 (14)	C7—N1—C6—C5	179.81 (17)
N2—C11—C12—C13	−128.3 (2)	C7—C8—C9—C10	−103.93 (18)
N2—C14—C15—C16	−130.4 (2)	C8—C1—C2—C3	−179.20 (15)
C1—C2—C3—O1	179.27 (15)	C8—C1—C6—N1	1.18 (17)
C1—C2—C3—C4	−1.5 (2)	C8—C1—C6—C5	−179.56 (14)
C1—C8—C9—C10	76.48 (19)	C8—C9—C10—N2	179.04 (12)
C2—C1—C6—N1	−178.59 (13)	C10—N2—C11—C12	61.28 (17)
C2—C1—C6—C5	0.7 (2)	C10—N2—C14—C15	−164.89 (14)
C2—C1—C8—C7	178.83 (16)	C11—N2—C10—C9	65.56 (16)
C2—C1—C8—C9	−1.5 (3)	C11—N2—C14—C15	65.76 (18)
C2—C3—C4—C5	1.4 (3)	C14—N2—C10—C9	−63.11 (17)
C3—C4—C5—C6	−0.2 (3)	C14—N2—C11—C12	−170.43 (13)
C4—C5—C6—N1	178.27 (16)	C17—O1—C3—C2	−13.9 (2)
C4—C5—C6—C1	−0.8 (2)	C17—O1—C3—C4	166.81 (16)
C6—N1—C7—C8	0.46 (19)	C19^i^—C19—C18—O3	14.9 (3)
C6—C1—C2—C3	0.5 (2)	C19^i^—C19—C18—O2	−165.31 (18)
C6—C1—C8—C7	−0.89 (17)		

Symmetry code: (i) −*x*, −*y*+1, −*z*.

### Hydrogen-bond geometry (Å, º)

**Table:**

*D*—H···*A*	*D*—H	H···*A*	*D*···*A*	*D*—H···*A*
N1—H1···O2^ii^	0.87 (1)	1.91 (1)	2.7729 (17)	175 (2)
N2—H2···O3	0.90 (1)	1.68 (1)	2.5669 (16)	171 (2)

Symmetry code: (ii) −*x*+1, −*y*+2, −*z*+1.
